# “That’s True Love:” Lived Experiences of Puerto Rican Perinatally HIV-Infected Youth within Their Families’ Context

**DOI:** 10.3390/ijerph13010007

**Published:** 2015-12-22

**Authors:** Georgina Silva-Suárez, Elena Bastida, Silvia E. Rabionet, Consuelo Beck-Sagué, Irma Febo, Carmen D. Zorrilla

**Affiliations:** 1 Department of Sociobehavioral and Administrative Pharmacy, Nova Southeastern University, 00926 San Juan, Puerto Rico; 2 Department of Health Promotion and Disease Prevention, Florida International University, Miami, FL 33199, USA; ebastida@fiu.edu (E.B.); cbecksag@fiu.edu (C.B.-S.); 3 Department of Sociobehavioral and Administrative Pharmacy, Nova Southeastern University, 33314, Fort Lauderdale, FL & Department of Social Science, School of Public Health, University of Puerto Rico, Medical Sciences Campus, 00921 San Juan, Puerto Rico; rabionet@nova.edu; 4 Department of Pediatrics, University of Puerto Rico, Medical Sciences Campus, 00921 San Juan, Puerto Rico; irma.febo2@upr.edu; 5 Department of Obstetrics and Gynecology, University of Puerto Rico, Medical Sciences Campus, 00921 San Juan, Puerto Rico; carmen.zorrilla@upr.edu

**Keywords:** adolescents, HIV/AIDS, death/bereavement, parent/child relations, family, qualitative

## Abstract

The burden of HIV affects not only HIV-infected patients but also their families and caregivers. It is also known that family support is crucial for people living with HIV. A qualitative study was conducted to explore the life experiences, within the family context, of perinatally HIV-infected (pHIV-I) youth in Puerto Rico. Twenty in-depth interviews were performed and audio-recorded. Within the family context, study participants experienced acceptance, love and support but also stigma and discrimination. They reported that family is an essential component in their lives and treatment. Losing one or both parents at a young age was considered more difficult than having HIV. Most participants who lost their parents lived with other family members. This was a challenging situation for both pHIV-I youth and their caregivers. Participants described their healthcare providers as part of their families and would like to keep in touch as they transition to adult care. Despite the challenges, participants expressed a desire to have children. Services targeted to this population should stress social support, incorporate family members into the medical process, provide special guidance and support while transitioning to adult care, and provide them with the latest information regarding HIV and reproductive options.

## 1. Introduction

Puerto Rico has shown evidence of major accomplishments in the prevention and treatment of HIV; the eradication of perinatal HIV transmission being one of the most significant. Puerto Rico has not reported a perinatal HIV transmission since 2011 [1,2]. Despite this success for future mother and children, there is still a generation of perinatally HIV-infected (pHIV-I) youth living with HIV on the island from which we can learn valuable lessons about how to respond to the life-long burden of a disease. 

It is well known that HIV not only affects the individual but also disrupts the foundation of the family structure [3,4,5,6]. Families of HIV-infected patients encounter stigma and social discrimination linked to the disease [7,8]. In some cases, the disease label may be applied to an entire family; in a village in Nigeria, for example, when a person acquires HIV infection, the villagers call the whole family an “AIDS family” [9]. Children of families affected by HIV struggle to understand why they are mistreated [7].

HIV-infected individuals and their families also face financial problems in addition to confronting the burdens of the disease. For example, in the Caribbean and worldwide, employers may test prospective and current employees, often without advising them that they are being tested or informing them of their results; this information is used to exclude HIV-infected persons from employment [10]. In China, employees reported to be fired because they tested HIV positive [9]. Moreover, the disease also has financial implications for those uninfected family members expected to provide support to the affected. Worldwide, families not only help the affected individual to cope with the disease but also provide financial and medical assistance and psychological support [9]. 

Family support is crucial for people living with HIV and can be critical to decision-making related to treatment-seeking and other situations [9]. Among adolescents living with HIV, family members and caregivers may be the ones who remind them to take their medication [11]. Among pHIV-I youth, family members assumed responsibility for their treatment when they were young children [12], and this support often continues into adolescence.

Among Latinos, close and supportive family relationships are important throughout the life span [13]. Maintaining a close relationship with family and viewing the family as a source of emotional and material support is known as *familismo* (for an example, see [14]). Puerto Ricans value their social relationships and consider family and friends as a source of support in their life trajectories [15]. 

This article explores the life experiences of Puerto Rican pHIV-I youth within a family context, focusing on the evolving nature of families and the diverse challenges faced by these youth and their kin. Participants’ narratives of their lived experiences enable us to examine diverse periods in their lives. In addition, these accounts should facilitate understanding the meanings and constructions they assign to “family.” Through their narrated experiences, we learn what they value the most, who they consider family, how they make sense of their life situation following a devastating blow in their lives, feelings about their family, and desires to start a family of their own.

## 2. Methods

This study used interpretative phenomenological analysis (IPA), a qualitative framework that seeks to explore how persons make sense of their everyday life experiences [16]. Phenomenological research seeks to examine an experience or phenomenon “the way it occurs and in its own terms” (p. 12, [16]). The IPA approach allowed researchers to gather the vivid recall of participants’ lived experiences within their family context, in addition to the meaning they ascribed to those experiences.

### 2.1. Sample Selection

Purposeful sampling was used to recruit study participants. This sampling method seeks to select information-rich cases “from which one can learn a great deal about issues of central importance to the purpose of the research” [17]. The in-depth interviews were conducted to collect participants’ life experiences. Eligibility requirements included: being current or former patients of the Pediatric HIV/AIDS Research Program; pHIV-I; aged 18–30 years, having knowledge of their HIV status; and willingness to share their life experiences.

The Pediatric HIV/AIDS Research Program’s staff assisted with the recruitment and in accordance with the Institutional Review Board (IRB) approval, participants and their parents or guardians (if the patient was under 21 years old) gave written informed consent.

### 2.2. Data Collection

An interview guide was developed by the first author. In keeping with IPA framework, the questions sought to elicit both the respondents’ experiences with certain phenomena and how they interpreted these phenomena. Later, the interview guide was assessed by a group of experts on perinatal HIV infection and piloted with a potential participant. No changes were made after conducting the pilot.

Twenty pHIV-I women and men between the ages of 18 to 30 years were recruited and interviewed. The interview lasted 45 to 90 min. Interviews were conducted in a non-judgmental, respectful environment at a sister clinic to the Pediatric HIV/AIDS Research Program that also provides services to people living with HIV. All interviews were conducted in Spanish, the native language of both study participants and the first author. After interview completion, each participant received a monetary incentive to cover transportation and other expenses associated with study participation.

All interviews were audio recorded, then transcribed by the first author. Transcripts were sanitized by removing any comments that may have compromised confidentiality. A graduate research assistant, immersed in Puerto Rican culture and proficient in English and Spanish, translated all interviews. The original Spanish text was used in conducting the qualitative analysis presented here, thus avoiding a third level of text interpretation, as indicated in qualitative procedures.

### 2.3. Data Analysis

Transcripts were examined individually. Following the steps proposed by Smith [16], the researchers read and re-read each of the transcripts establishing the participant as the focus of analysis and initial observations were recorded. In addition to the first author, transcripts were read by at least two other members of the research team and contributors to this paper. Passages from the interviews were then discussed with other team members in relation to situation and meaning construction, as suggested by the IPA approach.

The first exploratory stage of the analysis identified approximately 40 themes. These were narrowed down or consolidated according to the following criteria: (1) their recurrence within and across cases (how often they were mentioned); (2) their relevance to family context; (3) the relationship across themes.

**Figure 1:**
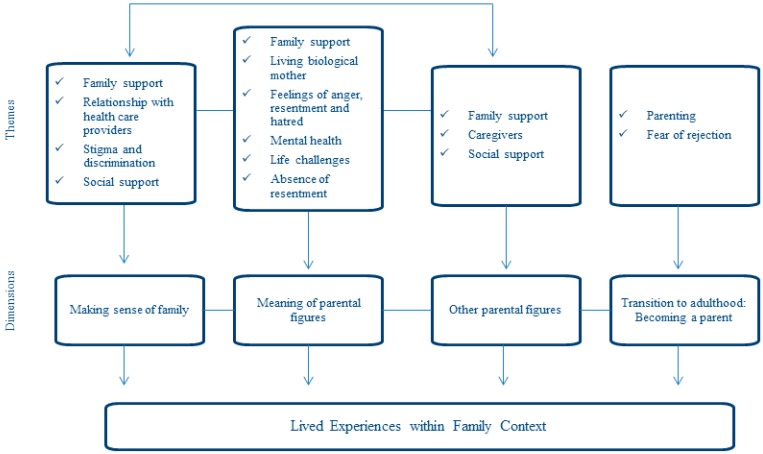
Patterns and connections among themes that emerge during phenomenological analysis.

To understand the meaning that participants constructed when talking and introspectively examining their family experiences and interactions, twelve interrelated and connected themes were selected. Figure 1 presents a diagram indicating how these eleven themes are not only interconnected, but fall within the four broader dimensions that gradually emerged from the narratives’ participants constructed when attempting to relate their lived experience within their family context. Each of these four dimensions is used below in organizing the data analysis and in illustrating the findings.

### 2.4. Multiple Coding (Inter-Rater Reliability)

In order to confirm the themes that emerged, 10 of 20 interview transcripts were assessed by two professionals, one in the area of human rights and the other in public health. Both identified major themes from the same 10 transcripts. The emerging themes were then cross-checked by each professional. When this procedure revealed discrepancies in topics, the coders clarified and discussed other possible interpretations, refined or created a new code or theme. NVIVO 10, a qualitative software, was used to facilitate the systematic processing of the information generated by in-depth interviews. 

## 3. Findings

### 3.1. Demographic Characteristics of the Study Population

Of the 20 study participants, eight were male and 12 were female. Participants ranged in age from 18–29 years (mean age = 22 years). Eighteen had completed high school, two had completed middle school and 10 were enrolled in some college program at the moment of the interview. Only one study participant was married, 16 were single and three were cohabitating. Eleven of them were working at the time of the interview. 

### 3.2. Making Sense of Family

While exploring the life experiences of pHIV-I youth, family emerged as a central presence and foundation in their lives. Four broad dimensions were particularly highlighted from participants’ narrated life experience related to family: (1) making sense of family; (2) the meaning of parental figure; (3) the roles of other parental figures; and (4) the transition to adulthood: becoming a parent. These topics were recurring themes in participants’ interpretation of their life experiences related to their families. Fragments of participants’ narratives were selected to support each of the themes; informants were given fictional names to protect their identities.

#### 3.2.1. Meaning of Family

When study participants spoke about the most significant people in their lives, all of them agreed that these were members of their family. Family meant everything to them, as expressed by Emma a 20-year old student: “My life. My family is my everything. Family is… (silence) everything for me, like a little part of you”.

Family is where they find love, trust and support. Family members are the people who have been with them during their most difficult situations in life, and they were therefore very thankful for the solidarity they received from family members.

#### 3.2.2. The “Dark Side” of Family

Even though most study participants said that family was the institution in which they found love, trust, and support, family experiences varied. Some study participants recall experiencing rejection from specific family members. Luz recalls how, when she was little, one of her great-aunts seemed to fear “contamination” whenever she visited their home: “My grandmother’s sister, whom we call “aunt,” would not eat our food or drink from a glass whenever she came to visit us”.

Kathy, a 21-year old self-employed woman, was visibly affected as she reconstructed how her sister told everyone about her diagnosis, disregarding her will or her feelings about the matter.

… Because she does not have it, she told others, because we have never gotten along well. [HAVE YOU TOLD HER HOW YOU FEEL?] The one who assumed that role, of talking to her, was mom, but she [her sister] still sort of treated her even more disrespectfully.

#### 3.2.3. Healthcare Providers as Family

When study participants talked about their health care providers, they described them as part of their extended families. Most respondents agree that those who have been taking care of them were also their family. Even though some recognized that medical visits and exams could be tiring, they also acknowledged the love and support they had received from their healthcare providers. Study participants said that not only did they help and support them but also supported their family.

The majority of them agreed that the clinical care they received has been good, but what they most appreciate is their providers’ affection and sincere dedication towards their wellness. Arturo shared his experiences at the Pediatric HIV/AIDS Research Program and his perception of what he thinks is most important in clinical care:

Their quality, the human quality that they show, they are very special to me. Very special to me… that is what I will miss the most, because I know that the medicine is there and any doctor can prescribe it. But it’s not only a medical issue, it’s that they care about me, they look after me… That is worth more than anything else.

Those already transitioned into adult care, acknowledged that adult care is very far from what they were used to in pediatric care. This is Esteban’s experience: “…the fact is that never again have I been treated like at [Pediatric HIV/AIDS Research Program]… When I go elsewhere to get care, it is not the same”.

They were thankful to have had great care during their developmental years, helping them cope with their life challenges. Mauricio narrated his experiences at the Pediatric HIV/AIDS Research Program:

… the doctors here, in project [Pediatric HIV/AIDS Research Program] they’ve provided me with great psychologists, great doctors who have helped me, you know, accept my condition. And always, since I was little, even when they have not told me what my condition was, they taught me to value myself, to not feel less than anyone. They taught me to be a confident person and so all those motivational words from the psychologists, from the doctors, well my family, also church groups… Aside from being health professionals, from being nurses or doctors, they’ve also been a helping hand, they’ve supported me... They’re like family to me.

### 3.3. Meaning of Parental Figures

Even though study participants said that family meant everything to them, the fact is that most families have experienced very difficult times. Losing family members, dealing with a potentially fatal illness, and taking care of a newborn, are only some of the situations the families of pHIV-I youths have had to cope with. Five study participants said that they had lost both parents due to HIV; another eight had lost at least one parent. Participants characterized the burden of losing one or both parents at a young age as a heavier weight than having HIV. This early loss irrevocably became the catalyst for numerous life challenges pHIV-I youths have had to address.

#### 3.3.1. Mother Figures

Six study participants considered themselves blessed because their parents are alive and they know many other perinatally HIV-infected youth who have not been as fortunate; several referred to their mothers as their “life.” All of them said that they love their mothers very much; they are proud of them and see them as role models and “fighters.” In addition, they recognized that sharing the same health condition and having an ally in their life journey made it more bearable. This is Emma’s experience, she considers herself blessed to have her mother around:

My mom… she’s my life, I love her, she’s always been there with me, always. [I feel] lucky to have her with me. To have her healthy. I am proud of her, because she really has overcome a lot, she’s a fighter.

When study participants talked about their fathers, their opinions and feelings varied considerably. Most spoke fondly about their mothers but not so about their fathers. This is often linked to the fact that they see them as the source of their HIV infection. 

#### 3.3.2. Feelings towards Their Fathers

Most of the study participants’ mothers were believed to have been infected by their partners—the study participants’ fathers—with injection drug use as the fathers’ route to HIV infection. 

Fernanda was one participant who learned that her HIV infection indirectly resulted from her father’s drug use:

There they told me that it was that my father used drugs and infected my mother who infected me, and that was all. I can’t deny that I feel a lot of resentment toward him even if he’s dead… but I can’t forgive him.

Some study participants said that their mothers were not necessarily aware of their fathers’ HIV infection; they therefore feel resentment towards the father. Isabel shared her feelings towards her father:

At present the person I hate is my dad. Because knowing that he had it and the condition he had, he didn’t care and he didn’t take care of my mom. [SO HE KNEW?] He knew, actually, before being with my mom; three or four women in the same area where my mom lived died because of him.

Luz, a 23-year old mother of one, expressed feelings of disgust and hatred towards her father, talking harshly about him. Other respondents said that forgiveness was not possible, because they blame their fathers for the loss of their mothers and for their own HIV infection:

When mom told me about what I had, and have, well I got upset with my father and, since then, I have never loved [him]. He is dead. He died at twenty-something... I’m glad he’s dead because otherwise I would kill him.

In this situation, the patients as well as their siblings and other family members suffered the consequence. One study participant said that faith in God helped her to forgive her father for transmitting the HIV to her mother and indirectly to her, but her sister could not and cannot handle the situation, expressing hatred toward him.

Several participants did not show any resentment towards either parent. For example, Alonso expressed compassion and understanding, saying that he cannot blame them but the circumstances; and that he is sure that his parents loved him.

It’s like I say, I know that if it wasn’t for her I wouldn’t have gotten it because she’s my mom and I know she loved me and my dad too; they loved me, you know, so I don’t feel like that… I don’t saddle them with blame. The circumstances are to blame, because everyone had their thing and not everyone is perfect and they made mistakes. So I can’t judge them because of that, nor will I say that they’re bad …At least they gave me life and until this day I’m pretty big and fat [both of us laugh]; I really don’t feel bad about this…

#### 3.3.3. Loss of Parents

The ages at which study participants lost their parents ranged between one and 16 years. Regardless of the age at which they lost them, the impact on their lives was profound. While some do not want to remember them, others yearn to be able to hear their mother’s voice at least once more. This is the case of Isabel, a 22-year old mother of two who also said that having HIV and overcoming the challenges and the burden of the disease would have been easier with her mother alive. Her interpretation of that experience is as follows:

My mother died the day of my 1st birthday. It’s very, very painful, because my mom was only 26 years old when she died, a girl. And not being able to, how do I say it, not being able to have her next to me, at the time I found out about the condition, and for her to help me, hear her voice, to know how she spoke, to feel her next to me, we would have gone through the same, we would have been able to understand each other so well. And not to have been able to live those experiences, well…

The families of two study participants told them that their first birthday was celebrated in the hospital so that their mothers could participate. Because of the death of their parents, some study participants concluded that those affected with HIV can die due to this condition; it was at that moment that they started to see HIV as a something to be feared. Some study participants sought psychological assistance to help them overcome the loss of their parents. Alonso, a 20-year old participant who lost his mother at a young age, did not worry about HIV at that time but rather found the loss of his mother devastating:

Yes, well, right, because, when I lost my mom, because of the condition, when I was five years old and she was 21. So that didn’t affect me at that point, it wasn’t about the condition itself. It was because I had lost my mom.

### 3.4. Other Parental Figures

#### 3.4.1. Adoptive Parents

Participants who were adopted expressed warm feelings towards their adoptive parents. They now acknowledge that taking care of an HIV infected infant was probably difficult, so they are very grateful for their adoptive parents who adopted them nevertheless. Victoria, an 18-year old student, relates how her mother adopted her even though her health condition was precarious at the time. She said that it was an act of “true love”:

That was true love, because not everyone does that, not everyone does that. And besides, I was very little, had the condition, and then I got very sick. I don’t know what I had but I was close to death. And when I was born I weighed 4 pounds and I was really tiny, I was 13” or something like that, I was very small, really tiny. And when she saw me, well, she fell in love and adopted me.

#### 3.4.2. Caregivers

Most study participants who lost their parents lived with other family members. This was a challenging situation for the caregivers. Not only did they have to deal with the HIV infection but also with the frustration and suffering of the study participants. Oftentimes; grandparents were the ones who assumed the responsibility for their care; a particularly difficult situation. Some were still grieving their daughters or sons; while they also had to assume responsibility for their grandchildren. For some, accepting the death of their own children and discovering that their grandchildren had an HIV diagnosis were difficult and very emotional situations to absorb. Most had to resume parenting their grandchildren at a later stage in their lives. Moreover; they had to learn how to manage institutional matters; such as school applications and medical care in order to obtain benefits for their grandchild. This was the situation of Marina; a 23-year old student:

Well, when I was very sick—you know, I was already [sick] at two months of age- my grandparents, who were always very close to my mother, were desperate. And when they took me to the hospital… and my grandmother was told (the diagnosis), well, she said she could not take it. She went into a depression, also because of my mother. My mother’s death was something that had a strong impact on them… But it was not just terrible for them… they did not know what to do in 1989 with a two-month old baby who was almost dying; the doctors told them “look, she is not likely to live long…” It was awful for them to accept that, and realize it, and try to live through it.

Caregivers also had to deal with rebellious acts on the part of study participants. Isabel shared how she started using drugs as a way to handle the difficulties she was going through, such as losing her mother, an uncle, enduring sexual molestation, and disliking her physical appearance due to the side effects of HIV medication. She later said she gave up drugs because of the suffering she was causing to her grandmother and the family who had been taking care of her since she was one year old:

I went through drug addiction. That was another experience I went through after I found out about my condition. I began to hang out a lot with a cousin and I began to learn about pot, cigarettes, heroin, crack... I did try it, and used it at least once. And I have to tell you that I became really crazy. But I have no regrets because I think that was a process I had to go through because of what had happened. Now, what is important is to change and be different and show my children that it is wrong.

### 3.5. Transition to Adulthood: Becoming a Parent

Most study participants agreed that they would like to be parents someday. Some said they were too young to take that step and also acknowledged that because of their HIV status they need to be more cautious than other peers. This is Emilia’s perception of becoming a mother: “It’s going to be somewhat difficult, because it is not going to be a normal process. I am going to have to be more cautious than any other mother...” (Emilia, 21 years old).

Six study participants were parents at the time of the interview. All agreed that being a parent was the most wonderful thing they have experienced. This is what Luz, a 23-year old mother of one, shared about being a mother: “It’s brutal. It’s good, but it is difficult, though good”.

Arturo also shares his perception of being a father: “Very satisfactory and very enriching. Very good, it really has been”.

All study participants who reported being a parent said that their children as well as their partners were not HIV infected. However, some study participants feared that their children could suffer HIV stigma and discrimination as a result of having an HIV-infected parent. Isabel’s fear extends well beyond this concern. She fears that if her kids are discriminated against because of her HIV infection, they will turn on her and judge or reject her because of that:

Even if they don’t have it, they may be rejected because they’ll think that they have it. Because there are a lot of ignorant people in this life. And […] if they get picked on, then they’ll go home and they’ll start judging me, and blame me for having been treated like that, and… and having to live through that experience scares me. It scares me…

## 4. Discussion

The meaning that the study participants ascribe to their family is linked to how the family fulfills its members’ needs. As they reconstruct their life experiences within the family context and daily interactions with family members, they emphasized their appreciation for the support they have received from them. As is the case among other cultural groups, most Puerto Rican families rely on family members as the first sources of support in adverse situations or even when facing ordinary aspects of daily life [15].

Family members usually know valuable information about each other; this information can be used to support and help them through life. However, on occasions this information can become a source of discomfort and suffering. Family interactions can be tinged with stigma [15]. One study conducted in Puerto Rico found stigma toward HIV positive individuals among family members [18]. Study participants shared their experiences of discrimination and breaches of confidentiality within their families.

Study participants reported receiving support and love from others sources, such their healthcare providers, which they refer to and consider an extension of family. In a study conducted in South Africa, researchers found that HIV/AIDS couselors and health-care providers were considered family members due to the support they provided to their patients [8]. Others research studies have shown that study participants attach much value to their relationship with their healthcare providers [11,19,20]. In agreement with the aforementioned research, most study participants expressed how they and their families received support, caring, affection, medical care, and love from their health professionals during their journey with HIV. These experiences led participants to consider their professional caregivers as “family.” Study participants expressed gratitude to these professionals and credited them for helping them to accept their HIV status and feel empowered and confident about themselves. Their healthcare providers have become a key source of social support for them.

Mothers are crucial support figures for study participants. They observed how their mothers deal and manage their own HIV-infection and also how they help them to cope with theirs. In a sense, the mother who shares her diagnosis becomes not only a role model but also a “socializing agent” who directly transmits to her child the knowledge and behaviors necessary in responding to this condition. 

In contrast, participants had a different, negative perception of their fathers. The life experiences of study participants within their families are context-dependent; their perceptions of HIV are also contingent on their environment. All study participants were born within the first decade of the HIV/AIDS epidemic (1983–1994); their views on their fathers could, therefore, be linked to that particular historical period. At the beginning of the epidemic, HIV/AIDS was associated with particular groups in society that were stigmatized prior to the epidemic such as homosexuals, intravenous drug users, and commercial sex workers [18]. These negative societal appraisals could explain why study participants talked about their mothers in complimentary terms and spoke unfavorably about their fathers. Most blamed the fathers for their mothers’ HIV infection. Thus, they portrayed their mothers as unwitting victims while their fathers received few if any favorable mention. This finding supports prior research. A research study found that, when children learned about their mothers’ HIV infection, their reactions varied from manifestations of emotional support and closeness to denial and anger [21]. In that same study, one participant expressed anger toward his father, the source of his mother’s HIV infection.

Perinatally HIV-infected youth in this study expressed that one of the most difficult challenges they had faced in life was the loss of one or both parents due to HIV infection. For orphaned participants, HIV infection became a secondary challenge. They reconstructed their experiences with depression after loss of their mothers; in some cases psychological help was needed to help them overcome the devastating experience. Losing one of their family members represented a major disruption in the family. When an HIV parent dies, the family may assume responsibility of raising the children of the deceased [22,23,24]. This situation is difficult not only for the children but also for the family, since they now must face the burden and stress of caregiving [9]. 

For some study participants, achieving stability and learning to live without their parents became a struggle. Some spoke of their rebellion at that point in time; others began using illicit drugs to cope with their life situation. As noted by most study participants, caregivers had to address their immediate emotional needs while also learning to monitor their care, medical visits, and their schooling. Healthcare providers were a source of support for their families in this process.

Participants acknowledged that their parents and caregivers had to deal with much more than just the HIV infection. One emerging situation was coping with the children’s loss of their parents. On losing their parents, these children concluded that HIV is a horrific illness that can rob them of their lives as it did their parents’ lives. Participants acknowledged that it was a difficult situation for them as well as for their caregivers. The latter, as study participants narrated and reconstructed their interactions with caregivers, were grieving because of the loss of their sons or daughters, while they also noted that they had to deal with a child who had just lost one or both parents due to the same health condition. They understood this as a process that took time, but eventually, the family managed to regain a new balance that enabled it to cope with the life event that had shaken its foundation. 

Study participants who were adopted said they felt blessed and expressed much admiration toward their adoptive parents. At a disruptive moment in their infancy, adoption gave these participants an opportunity to become part of a family unit where they found balance and harmony. 

Although study participants have suffered the impact of diverse challenges, their life course has continued to evolve. One of the most common and expected transition to adult life is becoming a parent. This is the case for pHIV-I youth as well. According to some study participants, one of their major life milestones was becoming a parent. They said that being a parent is one of the best things that had happened in their lives, and they expressed gratitude for having HIV negative children. Childbearing was considered a defining moment in the lives of study participants; they constructed it as a new beginning in which they were given the opportunity and gift to create a family of their own. 

## 5. Conclusions and Implications

The HIV epidemic has evolved with similar characteristics worldwide. Whether a country is developed or developing, the disease “disproportionately affects the poorest parts of the world” [12,25,26,27]. In the wealthiest countries, it primarily impacts the poorest areas and most socially marginalized people, including those who are homeless, substance users, and individuals from racial, sexual, and ethnic minorities [27,28]. In the United States, the highest prevalence of pediatric HIV infection is found in the poorest urban areas within ethnic minorities’ enclaves, which have historically suffered discrimination and racism [29]. Most of the striking racial and ethnic disparities seen in HIV prevalence in the urban US population disappear when controlling for spatial distribution in low-income neighborhoods, where more than 20% of residents live below the poverty level [30]. Similarities in the findings between this research study and others conducted around the world can be due to these social and demographic HIV epidemic characteristics shared worldwide. These similarities also indicate the prospect and desirability of establishing guidelines to address the needs of the pediatric population as they transition to adult care.

As the standard of care becomes patient-centered and as we promote shared-decision making to enhance health outcomes of those living with HIV, the experiences of one can become the contribution for many. Based on the reconstruction of participants lived experiences within the family context, some areas have been identified that need to be considered when dealing with this particular population. Notable among these is the hatred some study participants harbor toward their fathers. The desirability of exploring a more balanced, mature understanding of their fathers’ behaviors is a significant unmet need in the highly supportive care that these youth have received. The perspective that study participants have towards their mothers *vs.* their fathers and the effect of these opinions on their lives should be further investigated. Borchert and Rickabaugh found that mode of HIV transmission is a determinant factor that influences others’ perception of “personal control, positive affect, and willingness to help people with AIDS” (p. 664, [31]). Perception of individuals who acquired HIV through intravenous drug use was associated with less positive affect and less willingness to help [31]. Drug use is a complex social problem that in most cases affects the family system. A study conducted among incarcerated substance users in Georgia found that fathers were not an integral part in the lives of this population; most expressed anger and sadness for the fathers’ lack of involvement in their lives [32]. In addition, those incarcerated men who were fathers were not able to make a connection between their feelings towards their fathers and the possible pain they were causing to their own children, when they were almost replicating their father’s behaviors [32]. It would be worthwhile to study pHIV-I youths’ perceptions about their parents more deeply to better understand what influences their perceptions and to ascertain if substance use is a determinant factor in their perceptions.

A second area that was identified and found important to address is reproductive counseling and decision-making regarding family procreation. Six study participants expressed being parents at the time of the interview; however, none mentioned family planning, strategies for deferring or planning future pregnancies while exploring precautions to protect their partners. In the current study, only one study participant mentioned her intention for her and her future partner to be informed regarding reproductive alternatives. Participants also expressed a need to be better informed regarding new advances for HIV prevention such as pre-exposure prophylaxis (PrEP). Even when not placing HIV at the center of their lives, they acknowledged a need for education and information about their health condition as an important aspect of their wellbeing and quality of life. 

Currently, study participants are going through a major transition from pediatric care to adult care. When speaking of this situation in the context of their family situations and interactions, all emphasized that this transition would be a benefit if the adult care were to follow the Pediatric HIV/AIDS Research Program’s model. All also reiterated that whatever model is developed by adult treatment clinics, these need to be comprehensive and incorporate their families into the treatment plan. Time and again, their narratives emphasized their desire for a holistic approach that incorporates their families in all aspects of treatment. In their narratives there is a wistful, sentimental characterization of the Pediatric HIV/AIDS Research Program’s clinical services that might reflect in part a nostalgic recollection of the pediatric setting, while anticipating or recalling the less-supportive climate of the adult setting. 

Even though this study was not conducted to evaluate the Pediatric HIV/AIDS Research Program’s performance, the findings revealed that the work and services provided to the study population were highly valued and appreciated. Study participants were able to look beyond themselves and acknowledged that Pediatric HIV/AIDS Research Program’s staff helped them and their respective family members.

Puerto Rico has long-standing HIV research experience and has been the venue for groundbreaking research with HIV [23,33]. This is the first study that explored the lived experiences of a generation of pHIV-I youth in Puerto Rico. This study adds to previous research by providing a different perspective and capturing the thoughts, sentiments and feelings of a group of patients that were impacted by the outcomes of groundbreaking discoveries in Puerto Rico and worldwide. This study enabled us to learn a lot from this population and understand our pHIV-I youth better as well as allowing us to reflect on the services that they received. Through their narrations, we learned about the challenges and triumphs that they attributed to the health care services. The Pediatric HIV/AIDS Research Program not only impacted their medical situations but also became a source of social support and an extended family. In order to sustain and provide continuity, the adult care institutions throughout the Island could benefit from the findings reported.

The narratives of the life experiences of pHIV-I youth gathered in this study suggest an urgent need for comprehensive health services for this population in the context of Puerto Rico. Some study participants acknowledged that they needed psychological help to overcome and understand some of the difficult situations they encountered at some point in their lives in addition to their medical services. In addition, their narratives reveal that the love and support they receive from their family, friends, and health care providers help them overcome their life challenges. They expect adult health care to not only prepare them to face challenges relevant to their HIV condition but also to help them when other challenges emerge, for example, reproductive decisions.

## 6. Study Limitations

Due to the sample size and nature of the study, generalizations of study findings cannot be made. Participants’ family experiences are individual and context-dependent. Therefore, the life experiences of pHIV-I youth in the metropolitan area of Puerto Rico may not be the same as that of a youth in the rural area or on another Caribbean island.

## Appendix

Spanish quotes as appear in the manuscript:

– Mi vida. Mi familia es mi todo. Familia es… (silencio) todo para mí, como esa partecita de uno (Emma, 20 años).
– La hermana de mi abuela, que le decimos titi, ella cuando venía a visitarnos no quería coger de nuestra comida, no quería beber del mismo vaso (Luz, 23 años).
– Como [Ella] no lo tiene, se ponen a divulgarlo porque nunca nos hemos llevado bien [¿LE HAS DICHO COMO TE SIENTES?] La que tomaba ese papel de hablarle era mami, pero a mi mai como que muchas veces [su hermana] le faltó el respeto peor (Kathy, 21 años).
– La calidad, la calidad humano que son ellos. Ellos son muy especiales para mí. Muy especiales para mi….eso es lo más que voy a extrañar, porque yo sé que la medicina está y cualquier médico me puede recetar, pero no es cuestión médico, es que se preocupan por ti, te pregunten… eso vale más que todo (Arturo, 22 años).
– Realmente, nunca me han vuelto a tratarme como me tratan en [la clínica de VIH/SIDA de la Universidad de Puerto Rico]… cuando voy a otra institución a tratarme, no es lo mismo (Esteban, 26 años).
– Bueno, porque aquí los doctores, en [la clínica de VIH/SIDA de la UPR] me han tenido unos muy buenos psicólogos, muy buenos doctores que me han ayudado, a tu sabes, a aceptar mi condición. Y siempre, desde pequeño, aunque no me decían cual era la condición, pero si me enseñaron a valorarme a no sentirme menos que nadie. Me enseñaron a ser una persona segura y entonces todas esas palabras de motivación de parte de los psicólogos, de los doctores, pues de mi familia, también de grupos de la iglesia… Aparte de ser profesionales de la salud, de ser enfermeros o médicos, pues han sido también una mano amiga, han sido una mano amiga, me han apoyado y han sido más bien para mí como parte de una familia. Son como una familia para mí (Mauricio, 19 años).
– Mi mamá…ella es mi vida, yo a ella la amo, ella siempre ha estado ahí conmigo, siempre. [Me siento] afortunada de tenerla conmigo. Tenerla sanita. Estoy orgullosa de ella, porque ella sí ha luchado mucho, una luchadora (Emma, 20 años).
– Ahí me explicaron que era que papi usaba drogas y contagió a mami y que mami me contagió a mí, y así fue todo. No te voy a negar que siento mucho rencor hacia él aunque está muerto… pero no lo puedo perdonar (Fernanda, 29 años).
– En estos momentos a quien yo odio es a mi papá. Porque el sabiendo lo que tenía, y la situación en la que él estaba no le importó y no cuidó a mi mama. [O SEA EL LO SABIA] Él lo sabía, de hecho, antes de estar con mi mamá, por causa de él ya habían muerto 3 o 4 mujeres de la misma área de donde vivía mi mamá (Isabel, 22 años).
– Cuando mami me explicó por qué fue que lo tuve, lo tengo, pues me molesté con mi padre y desde entonces nunca [lo] he querido, él está muerto, él se murió a los veinti algo... Que bueno que está muerto porque si no,0 lo mato (Luz, 23 años).
– Es como yo digo, yo sé que si hubiese sido por ella no lo hubiese tenido porque es mi Mai y yo sé que ella me quería y mi Pai también, ellos me aman, tu sabes que no me siento así… yo no los pongo como culpables. Culpables la circunstancias porque cada cual tiene su y no todo el mundo es perfecto y ellos tuvieron sus errores, pues, no puedo juzgarlos por eso, no me voy a poner a decir que ellos son malos, son esto, porque por lo menos me dieron la vida y hasta el día de hoy estoy bastante grande y gordo [carcajadas de ambos] de verdad que yo por lo menos no me siento mal así… (Alonso, 20 años).
– Mi mamá falleció el mismo día de mi primer año. Es bien, bien doloroso, porque pues mi mamá a penas lo que tenía era 26 años cuando murió, una nena. Y el no poder, como te digo, no poder tenerla al lado, en los momentos que pues me enteré de la condición, y que pues me ayudara, escucharle la voz, saber cómo hablaba, el poderla sentir al lado mío, hubiéramos pasado lo mismo, nos hubiéramos podido entender tanto y el no poder haber vivido esas experiencias pues… (Isabel, 22 años).
– Si, bueno, verdad, porque, cuando yo perdí a mi Mai’, por lo de la condición, cuando yo tenía 5 años y ella tenía 21. Eso a mí no me afectaba en ese momento, no era por na’ de la condición, en sí. En sí era como yo perdí a mi Maí. (Alonso, 20 años).
– Que eso era amor de verdad, porque no todo el mundo hace eso, no todo el mundo hace eso. Y más yo, yo era chiquita, tenía la condición y después me dio, no sé qué me dio pero estaba a punto de morirme. Yo era, yo nací, pesando 4 libras, yo era una cosa bien chiquita, media 13” o algo así, yo era bien chiquitita, bien chiquitita. Y ella al verme, pues se enamoró de mí y pues me adoptó. (Victoria, 18 años).
– Pues, yo estaba tan enfermita, tu sabes, yo a los dos meses de nacidas estaba [enferma]… mis abuelos siempre fueron muy cercanos con mi mamá, estaban en desespero. Y cuando me llevaron al hospital… a mi abuela le dijeron mi [diagnostico], ella no pudo [manejarlo]. Ella cayó en depresión, también por mi mamá. Al mi mamá fallecer, fue algo bien impactante para ellos… Pero [no sólo] fue impactante para ellos… no sabían que hacer en el 89 con una bebe de dos meses muriéndose, como quien dice. Los doctores le decían “mira ella no va a durar mucho.” Fue algo bien fuerte para ellos aceptar y reconocer y tratar de “lived trhough it” (Marina, 23 años).
– Yo pasé por la drogadicción. Esa fue otra experiencia, que después que yo me enteré de mi condición. Me empecé a juntar mucho con una prima y empecé a conocer lo que era la marihuana, el cigarrillo, el perico, el crack. Todo eso lo probé, porque no puedo decir que me metí porque si no no estuviera aquí. Pero si lo probé, llegué a utilizarlo aunque sea una vez. Y te digo, yo me volví bien loquita, pero no me arrepiento porque creo que eso fue un proceso que tuve que vivir por lo que pasó. Ahora lo que importa es hacer el cambio y la diferencia y demostrarle a mis hijos que eso está mal (Isabel, 22 años).
– Va a ser un poco difícil, porque no va a ser como un proceso normal. Voy a tener que estar más precavida que cualquier otra madre… (Emilia, 21 años).
– [Hablando de la experiencia de ser madre] Brutal. Es buena, es difícil, pero es bueno (Luz, 23 años).
– [Hablando de la experiencia de ser padre] Muy satisfactoria y enriquecedora. Muy buena de verdad (Arturo, 22 años).
– Aunque no lo tengan van a quererlos discriminar, porque van a pensar que lo tienen. Porque hay mucha gente ignorante en esta vida. Y… si los pegan a relajar, que ellos lleguen a casa y me peguen a juzgar, que por culpa tuya me están tratando así, que…y tener que vivir es experiencia me da miedo. Me da miedo… (Isabel, 22 años).
